# The Penn State Worry Questionnaire Is Invariant Across Age Groups

**DOI:** 10.1093/geroni/igaa057.1198

**Published:** 2020-12-16

**Authors:** Robert Intrieri, Paige Goodwin

**Affiliations:** Western Illinois University, MACOMB, Illinois, United States

## Abstract

Prevalence of GAD is between 3 to 5% with onset in the early to mid-twenties (Kessler et al. 2009). The Penn State Worry Questionnaire (PSWQ) is a 16-item self-report instrument assessing generalized anxiety symptoms (Meyers, et al., 1990; Molina & Borkovec, 1994). Brown (2003) and Olatunji et al. (2007) conducted Confirmatory Factor Analyses identifying a two factor model of Worry Engagement and Absence of Worry. No published studies have examined the factor structure of the PSWQ across age groups. The current study presents data from 612 people across three groups: 221 young adults (Mage = 19.31, SD = 1.21), 283 middle-age adults (Mage = 48.27, SD = 5.13), and 108 older adults (Mage = 72.95, SD = 7.19). An ordinal confirmatory factor analysis (CFA) using robust weighted least squares (WLSMV) tested for invariance across groups. Results showed CFI/TLI values of .983/.981, 984/.983, and .981/.984 for Configural (CI), Metric (MI), and Scalar (SI) models. The RMSEA for CI, MI, and SI models was .064, .061, and .059. Based upon Cheung and Rensvold (2002), Sass (2011), and Chen (2007), a cutoff of ΔCFI ≥ 0.01 was established as evidence of invariance. The ΔCFI between CI and MI models was < .01 so analysis continued with the SI test. The ΔCFI between MI and SI models was < 0.01 and did not justify rejection of the null hypothesis. These analyses suggest PSWQ scores are valid across age groups and provide additional support for the multidimensional nature of the PSWQ.

